# Targeted Therapies in Small Cell Lung Cancer: From Old Failures to Novel Therapeutic Strategies

**DOI:** 10.3390/ijms24108883

**Published:** 2023-05-17

**Authors:** Massimiliano Cani, Valerio Maria Napoli, Edoardo Garbo, Giorgia Ferrari, Benedetta Del Rio, Silvia Novello, Francesco Passiglia

**Affiliations:** Department of Oncology, University of Turin, San Luigi Hospital, 10043 Orbassano, TO, Italy; massimiliano.cani@unito.it (M.C.); valeriomaria.napoli@gmail.com (V.M.N.); edoardo.garbo@unito.it (E.G.); giorgia.ferrari@unito.it (G.F.); benedetta.delrio@unito.it (B.D.R.); silvia.novello@unito.it (S.N.)

**Keywords:** targeted therapies, small cell lung cancer, SCLC

## Abstract

The clinical management of small cell lung cancer (SCLC) treatment remains a major challenge for thoracic oncologists, with very few therapeutic advances significantly impacting patients’ survival. The recent introduction of immunotherapy in the clinical setting produced a marginal benefit for a limited subset of metastatic patients, while the therapeutic scenario for relapsing extended-disease small cell lung cancers (ED-SCLCs) remains almost deserted. Recent efforts clarified the molecular features of this disease, leading to the identification of key signalling pathways which may serve as potential targets for clinical use. Despite the large number of molecules tested and the numerous therapeutic failures, some targeted therapies have recently shown interesting preliminary results. In this review, we describe the main molecular pathways involved in SCLC development/progression and provide an updated summary of the targeted therapies currently under investigation in SCLC patients.

## 1. Introduction

Small cell lung cancer (SCLC) is a high-grade neuroendocrine tumour accounting for 15% of lung malignant neoplasms. Lung neuroendocrine tumours have been categorised as a single group of neoplasms since 2015, while the most recent 2021 World Health Organization (WHO) classification includes low-grade typical carcinoid (TC), intermediate atypical carcinoid (AC) and high-grade subtypes, such as small cell lung cancer (SCLC) and large cell neuroendocrine cancers (LCNEC) [1,2]. Globally, 250,000 new diagnoses and 200,000 deaths from SCLC are expected every year, with a higher prevalence in high-income countries, especially among men [3]. Over the latest 30 years, SCLC incidence decreased in the overall US population as a result of tobacco attitude reduction, thus reinforcing the strong link between tobacco consumption and SCLC occurrence. Recently a higher proportion of diagnoses have been reported among female and elderly patients, increasing from 23% in 1975 to 44% in 2010 [4]. Beyond tobacco smoking history [5], other risk factors include both air pollution and radon exposure, even if the evidence is still weak [3]. Although lung cancer screening programmes by low-dose computed tomography (CT) demonstrated a significant reduction of lung cancer-related mortality in the high-risk smoking population, no benefit was proven in SCLC disease [6,7,8]. Based on 1983–2012 data analysis, the median overall survival (OS) is seven months, with 70% of patients diagnosed at the metastatic stage. Even if a good response to upfront chemotherapy generally occurs in the majority of SCLC patients, this disease is characterised by a poor prognosis, aggressive behaviour and a fast doubling time [9]. Lately, the increasing development of new drugs seems to barely increase the life expectancy of SCLC patients, even if enrolment in clinical trials remains an important issue. Indeed platinum-etoposide represents the backbone chemotherapy combination for the first-line treatment of metastatic disease, while the recent results of both IMpower133 and CASPIAN randomised studies supported the association of immune checkpoints inhibitors (ICIs), atezolizumab or durvalumab, showing a significant improvement of both progression-free survival (PFS) and OS as compared to chemotherapy alone [10,11]. Differently from the first-line setting, no major advances have been made in pre-treated patients, with either platinum-doublets rechallenge, or single-agent chemotherapy still considered the most effective therapeutic options to be offered to our patients at the time of disease relapse [12,13]. Several clinical trials explored the potential role of immune checkpoints inhibitors in this setting, mostly leading to disappointing results. In detail, the phase 1/2 trial, CHECKMATE 032 study, investigated either nivolumab alone or combined with ipilimumab in PD-L1 unselected, relapsed SCLC patients. In this basket trial, the overall response rate (ORR) resulted in 11.6% and 21.9% for nivolumab alone and in combination with ipilimumab, respectively. Despite such a difference, the 12–24 months OS rates were similar between the two arms, while increased toxicity was associated with the combination therapy, including G3–G4 adverse events (AEs) rates of 37.5% vs. 12.9%, respectively [14]. Based on this study’s results, in 2018, the Food and Drug Administration (FDA) granted accelerated approval for Nivolumab as a third-line therapy for relapsing extended-disease small cell lung cancer (ED-SCLC) patients. In the same setting, the randomized phase III Checkmate 331 trial evaluated nivolumab vs. topotecan or amrubicin in PD-L1 unselected patients, showing a median OS of 7.5 months for nivolumab compared to 8.4 months for chemotherapy (Hazard ratio, HR 0.86, 95% CI: 0.72–1.04, *p* = 0.11) thus failing to reach the primary endpoint of the study. Of note, nivolumab monotherapy resulted safer than chemotherapy with G3–G4 AEs of 14% vs. 73% [15]. Pembrolizumab efficacy as third-line monotherapy in relapsed SCLC patients was otherwise evaluated in the phase Ib KEYNOTE-028 as well as in the phase II KEYNOTE-158 trials [16,17], showing an ORR of 33.3% and a mOS of 9.7 months and an ORR of 18.7% and a mOS of 8.7 months, respectively. A subsequent pooled analysis of both studies confirmed these findings, showing an ORR of 19.3% and a median OS of 7.7 months, supporting the FDA approval of pembrolizumab in this setting regardless of PD-L1 expression. Despite these encouraging results, in 2021, confirmatory phase 3 clinical trials results did not demonstrate an advantage in terms of OS for both pembrolizumab and nivolumab as compared to standard chemotherapy in pre-treated SCLC patients, leading FDA to withdraw such specific therapeutic indications in the relapsed setting [15]. In this challenging scenario, numerous clinical trials are in progress with the aim of identifying specific molecular features and potentially effective targeted therapies for SCLC patients. In this review, we describe the main molecular pathways involved in SCLC development/progression and provide an updated summary of the targeted therapies currently under investigation in SCLC patients.

## 2. Molecular Landscape of SCLC

### 2.1. Signalling Pathways and Molecular Alterations

**Figure 1:**
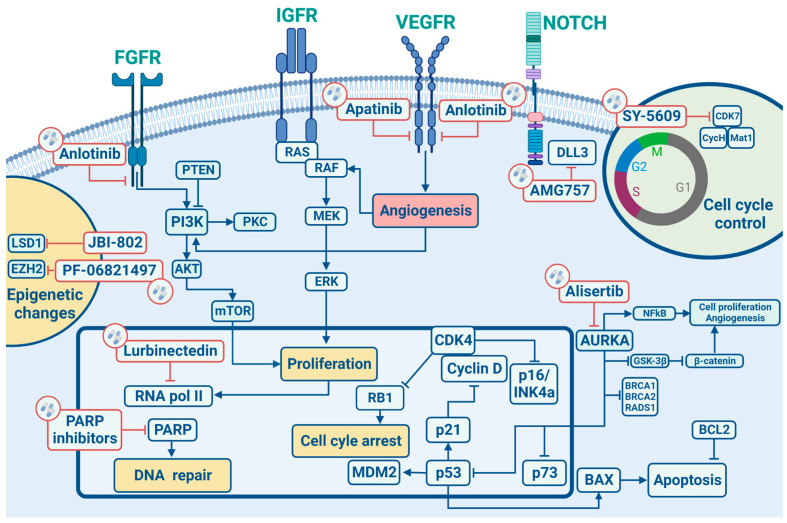
Signalling pathways and main targeted therapies in SCLC.

In this section, we will describe the main cell signalling pathways implicated in the development/progression of SCLC (Figure 1) by elucidating in the following sections their potential therapeutic implications.

Similarly to other epithelial tumours, SCLC is characterized by several chromosomal aberrations, including a great number of chromosomal deletions with recurrent losses at 3p, 5q, 13q and 17p regions, which are actually linked to tumour suppressor genes, as well as copy gains at 1p, 2p, 3q, 5p, 8q and 19p, encoding for well-studied oncogenes, such as MYC and KRAS, both highly correlated to tumorigenesis. Particularly, allele loss on chromosome 3p has been reported with a frequency greater than 90% in SCLC, likely representing an early molecular event driving lung tumorigenesis processes [18]. A recent large real-world data analysis revealed a potential positive prognostic role for gene amplifications on 4q12 (1.1%). This region encodes for VEGFR2, PDGFRA and KIT, receptor tyrosine kinase whose mutations have already been correlated to better outcomes with an improved mOS (67.9 months in the case of VEGFR2 and PDGFRA and 24.0 months for KIT) [19].

Although chromosomal aberrations are the most frequent molecular alterations detected in SCLC, another important role in the pathogenesis of this disease is played by RNA-dependent DNA polymerase, which restores the short regions of DNA called telomeres, normally shortened after repeated cell mitosis. In terminally differentiated cells, telomerase activity is usually suppressed, but more than 98% of SCLCs harbour an up-regulation of hTR (a telomerase RNA subunit) and a higher telomerase activity promoting tumour cell survival [20].

Tumour suppressor genes have great importance in SCLC tumorigenesis, and the loss of both TP53 and RB1 have been found, respectively, in 100% and 93% of SCLC patients. The p53 protein works as a down regulator of cellular proliferation by targeting downstream genes involved in cell cycle arrest (G1 and G2), DNA repair (GADD45) and apoptosis (BAX) [21]. Similarly, RB is implicated in cell cycle control by regulating the G1/S transition phase by the mediation of E2F1, E2F2 and E2F3 transcription factors. Indeed the cyclin D1/CDK4 complex, by phosphorylating RB, releases E2F, allowing its activation and transition to the S cell cycle phase [22]. In this context, the most known cell cycle regulator in SCLC is CDK7, an important regulator of cell-cycle progression. CDK7 works as the catalytic core of the CDK-activating kinase (CAK) complex and turns into the active form by binding to Cyclin H and Mat1. The trimeric CAK complex switches on several central cell-cycle CDKs by phosphorylation. A selective CDK7 inhibitor, YKL-5-12, was tested in association with anti-topoisomerase I, topotecan and ICIs, revealing that CDK7 inhibition predominately destroy cell-cycle progression and induces DNA replication stress and genomic instability in SCLC cells, promoting also immuno-response signalling. Combining YKL-5-124 with anti-PD-1 showed a significant survival benefit in multiple highly aggressive SCLC murine models, providing a rationale for combination regimens.

Not only tumour suppressor genes but also non-receptor oncogenes are involved in the SCLC onset. Bcl-2 is a member of a protein family that regulates cell death and other key cellular processes like apoptosis, necrosis and autophagy. Bcl-2 up-regulation was found in 75–95% of SCLC and, due to its peculiar biological mechanism, is involved in tumorigenesis, as already proven for other malignancies [23]. MYC genes family encodes the nuclear DNA binding proteins, c-MYC, N-MYC and L-MYC, which work as transcription factors regulating cellular proliferation, apoptosis, and differentiation, thus explaining its role in tumorigenesis [24]. In detail, MYC activation was reported in 18–31% of SCLC and has also been correlated to worse patient survival [21].

Many other intracellular signalling pathways linked to SCLC carcinogenesis have been identified, and among these, the most relevant are represented by PI3K/AKT/m TOR and PTEN pathways. In this regard, phosphorylated AKT was found in almost 70% of SCLCs [25], and mTOR, S6K1 and phosphorylated 4EBP1 protein expression is higher in SCLC cells compared to type II normal epithelial cells [26]. Such alterations have been linked to the cells’ growth, survival, and chemotherapy resistance, and more recently, correlated to brain metastases occurrence in SCLC [19].

A recent real-world data study comprising 3600 cases showed a more complex mutational landscape. Beyond the already known mutations involving both RB1 and TP53 genes, the authors identified an increased mutational rate of PTEN (9.9%), PI3KCA (5.6%), EGFR (3.4%), KRAS (3.3%) and NF1 (3.3%) genes, compared to previous datasets. Of note, some unknown mutational alterations were identified: 3% of all SCLC cases harboured Kelch like ECH Associated Protein 1 (KEAP1) inactivating mutations, thus suggesting a role of this tumour suppressor gene also in SCLC occurrence [19].

The tumorigenesis process in SCLC has also been linked to abnormal activities of tyrosine kinase receptors (TKR). In more detail, TKRs are involved in different cell signalling pathways such as cellular proliferation, migration and survival, thus emerging as potential therapeutic targets in SCLC, and c-Kit is a member of the PDGF/c-Kit tyrosine kinase receptor family. Upon binding of its ligand, the stem cell factor (SCF), cell growth and differentiation process are carried on through activation of the JAK-STAT, PI3K and MAP kinase pathways, thus contributing to tumorigenesis. The c-Kit expression has been reported in 79–88% of SCLC cell lines, while both c-kit and SCF expression have been demonstrated in 57–76% of SCLC cell lines [27]. Likewise, c-MET is another important TKR in SCLC; when activated by its ligand, hepatocyte growth factor/scatter factor (HGF/SF) is indeed able to modulate downstream molecules such as growth factor receptor protein 2 (Grb2), the p85 subunit of PI3K, STAT3 and Grb2 Associated Binder-1 (Gab1), leading to proliferation, survival, motility, invasion of the extracellular matrix and tubules formation. Overexpression and amplification of c-MET were shown in SCLC, and higher levels of HGF have been related to a worse disease prognosis [21]. The insulin-like growth factor receptor (IGF-1R), a member of the insulin receptor subclass of tyrosine kinase receptors, following activation by binding IGF-1 and IGF-2, promotes mitogenic, anti-apoptotic and transforming activities [28]. Protein levels of IGF-1 are elevated in over 95% of SCLCs, and furthermore, IGF-1R promoting the PI3K-AKT pathway has been correlated to tumour growth and chemotherapy resistance mechanisms. The tyrosine kinases fibroblast growth factor receptor family has four different isoforms (FGFR 1-4). Upon binding of fibroblast growth factors (FGFs), the receptor interacts with numerous signalling proteins and promotes Ras/Raf/MEK/Erk1,2 and PI3K-AKT signalling pathways [29]. Increased levels of FGF-2 in SCLC patients’ blood were correlated with higher angiogenesis and worse clinical outcomes; in the end, it has been reported that FGF-2 stimulates SCLC growth and chemotherapeutic drug resistance [30]. The vascular endothelial growth factor (VEGF) family comprises VEGF A-E forms and their three relative VEGF receptors (VEGFR 1-3). The VEGF signalling pathway leads to cell proliferation, migration and invasion of endothelial cells, thus mediating tumour angiogenesis. Increased levels of VEGF were found in patients with SCLC, and their levels were associated with tumour stage, disease progression, chemotherapy resistance and worse clinical outcomes. Inhibiting the VEGF/VEGFR signalling pathway may be an effective therapeutic strategy as reported in many other malignancies. Recently, good outcomes were reported in two phase II clinical trials in patients with extensive stage (ES)-SCLC when bevacizumab was added to first-line treatment followed by maintenance of bevacizumab itself [31]. Of note, Sivakumar et al. have recently found a potential correlation between gene amplification at 4p12 and increased OS in SCLC patients. This region encodes for VEGFR2, PDGFRA and c-Kit genes, suggesting a complex synergistic interaction between receptor tyrosine kinases and downstream pathways [19].

Developmental pathways, like Hedgehog, Notch and Wnt ones, regulate stem cell self-renewal. If abnormally activated, they can lead to neoplastic proliferation, representing an early event in tumorigenesis [32]. SCLC have a characteristic neuroendocrine phenotype, expressing neural and endocrine markers, such as synaptophysin, chromogranin A and CD-56 promoted by Notch and Hedgehog signalling pathways [33]. SCLC is strictly related to the Notch and Hedgehog signalling aberrations [34], and targeting these pathways may lead to more durable treatments. In detail, Notch signalling controls differentiation, development and cell destiny in a variety of contexts: overexpression of Notch receptors causes cell cycle arrest and stops growth inhibition of SCLC [35]. Therefore, promoting the Notch 1 pathway can be an effective therapeutic strategy in SCLC. A famous inhibitory Notch ligand is Delta-like ligand 3 (DLL 3): it is highly expressed in SCLC and is emerging as a promising molecular target for novel targeted drugs. Wnt proteins comprise a family of 19 secreted molecules with different expression patterns and a range of functions, including proliferation, differentiation, survival, apoptosis and cell motility [36]. During lung genesis, specific Wnt signalling is needed for normal epithelial-mesenchymal interactions. When Wnt pathways are deregulated, neoplastic events may occur. In NSCLC specimens, Wnt proteins (such as Wnt1 and Wnt 2) are overexpressed, and Wnt regulators (such as WIF) are down-regulated; thus, targeting this pathway can be otherwise a good strategy to obtain tumour control.

Cell surface markers have also been taken into account in SCLC tumorigenesis. The neural cell adhesion molecule CD56 is an isoform encoded by the NCAM gene and is related to the immunoglobulin family and controls neuroendocrine cell growth, migration and differentiation. NCAM is found in almost 100% of SCLC cells [37]. Although it is also expressed in other cells like natural killer cells, neuroendocrine glands, central and peripheral nervous systems, and cardiomyocytes, it has been considered a target for anti-cancer therapies.

Epigenetic changes physiologically occur in normal cells in order to control phenotype’s expression without DNA sequence changes. Methylation and histone modification processes involving the Zeste Homolog 1 or 2 (EZH1/2) enhancer usually play a prominent role as part of the polycomb repressive complex 2 (PRC2) transcription regulator. It has been shown that an EZH2 over-expression can lead to subsequent up-regulation of targeted genes involved in SCLC tumorigenesis as ASCL1, suggesting that EZH2 targeting could represent a promising therapeutic strategy in this setting [38,39]. Another important role is played by Schlafen family member 11 (SLFN11), which seems to be a predictor of response to DNA-interfering agents such as topoisomerase I and II inhibitors, platinum, and poly ADP-ribose polymerase (PARP) inhibitors [36].

The high mutational burden of SCLC is linked with the association of this disease with heavy tobacco exposure. This is the reason why in this context, DNA Damage Repair (DDR) pathway and cell cycle control are so important [5]. The loss of cell cycle checkpoint controls caused by the inactivation of RB1 and TP53 increases susceptibility to DNA damage and the therapeutic targeting of central DDR mediators, such as PARP, checkpoint kinase 1 (CHK1), Ataxia telangiectasia and RAD3-related protein (ATR), Ataxia telangiectasia mutated (ATM), and WEE1, has been recently investigated in SCLC as it leads to tumour cells death by genomic instability. Preclinical studies showed an increased response to anti-PD1/PDL1 drugs by blocking the WEE1 pathway in SCLC models. Indeed the inhibition of WEE1 signalling promoted G2/M cell cycle arrest, leading to the activation of the STING-TBK1-IRF3 pathway and increased concentrations of type I interferons (IFN; IFN-α and IFN-β) and other pro-inflammatory cytokines. Blocking the WEE1 pathway may help to empower tumour immunogenicity and potentiate the effects of immune checkpoint inhibitors, as suggested by the association of selective, small molecule WEE1 inhibitor, adavosertib (AZD1775) and PD-L1 inhibitors, leading to tumour regression in murine models of SCLC [40].

### 2.2. Emerging Molecular Classification

SCLC has been historically considered a unique molecular entity, while recent studies have demonstrated a heterogeneous molecular background allowing us to identify different disease subsets predicting variable responses to the available treatments [41]. To explore this in more detail, Gay et al. revealed the role of the Achaete-scute homologue 1 (ASCL1 or ASH1) and the neurogenic differentiation factor 1 (NeuroD1) in the neuroendocrine cell differentiation processes by activating specific genes like insulinoma-associated protein-1 (INSM1), but also MYCL1, MYC, RET, SOX2, BCL2 and NFIB genes [42]. Therefore SCLC cells harbouring ASCL1 and NeuroD1 have been associated with a pure neuroendocrine differentiation proven by the detection of higher levels of chromogranin A and synaptophysin and were classified as two different SCLC molecular subsets, respectively named group A and N. Due to the sporadic occurrence of non-neuroendocrine cells in SCLC, a third molecular subset was identified, characterized by the overexpression of TRPM5, GFI1B, SOX9, CHAT, POU2F3, ASCL2, AVL genes and RE1 silencing transcription factor (REST) which is a repressor of neuroendocrine genes thus confirming the non-neuroendocrine nature of this group (group P) [43]. Another group (I), characterized by the absence of all previous molecular biomarkers and the identification of inflammatory features, has been identified, predicting higher susceptibility to ICIs. Group I is characterised by overexpression of the RB1 gene, as well as HLAs and other antigen-presenting factors genes, besides PD-L1, PD1, CD80, CD86, CD38 and TIGIT [43]. Interestingly, recent studies revealed a possible dynamic evolution of SCLC subtypes from either A or P to I subgroups as a result of resistance occurrence following platinum-based chemotherapy [44,45]. Furthermore, subgroup analyses of randomized clinical studies have recently suggested an increased OS benefit to the ICI atezolizumab in the I group as compared to the other molecular subset (HR of 0.56 (95% CI: 0.321–0.998)) [43]. Additionally, the same group seems to be particularly sensitive also to Bruton’s tyrosine kinase (BTK) inhibitors like ibrutinib. Conversely, in vitro, studies have shown that subtype N can benefit from Aurora kinase (AURK) inhibitors. Meanwhile, subtype A is particularly sensitive to B-cell lymphoma 2 (BCL2) inhibitors. Platinum-based chemotherapy seems to be more effective in P subtype cell line models (*p* = 0.06); meanwhile, it appeared to be less effective in groups N and I [43].

Real-world data can further enrich this complex scenario by elucidating the mutational background of SCLC and thus providing additional insight into the molecular classification of this disease. In this regard, Sivakumar et al. have recently identified new molecular SCLC features by examining 3600 SCLC cases with FoundationOne^®^ or FoundationOne^®^CDx assays. Both TP53 and RB1 gene alterations were detected in 91.6% and 73.5% of cases, respectively, in line with previous data. Interestingly, authors identified unexpected TP53 and/or RB1 negative SCLCs and peculiar STK11 mutations, suggesting a new classification of SCLC including three different molecular subgroups: RB1 and/or TP53 wild-type SCLCs, STK11 mutated SCLCs, and finally, those SCLCs derived from NSCLC and characterized by peculiar driven mutations such as EGFR [19]. In detail, for RB1 (20.8%), TP53 (2.7%) or both (5.5%) wild-type tumours, authors hypothesized a different inhibitory mechanism on these crucial factors. Proteins of Human Papilloma Virus (HPV) such as E6/E7 are well-known inhibitory factors for both p53 and RB [46] and were identified in 87 cases of SCLCs tested in this study. Noteworthy, only 1.8% of TP53/RB1 mutated SCLC were HPV+ compared to 12.7% of TP53 and/or RB1 WT cases, thus reinforcing a possible correlation between SCLC and HPV infection. Of note, younger patients showed lower TP53 and RB alterations compared to the entire cohort (77.0% vs. 92.6% for TP53, *p* < 0.0001 and 60.7% vs. 74.4% for RB1, *p* < 0.0001), and similar data were also reported in the African ancestry population. The second group is otherwise identified by STK11 mutations, whose role has already been described in NSCLC. In the mentioned dataset, authors identified only 1.7% of STK11 mutated SCLCs; noteworthy, these tumours were enriched in both KRAS (3.3%) and KEAP1 (3%) mutations, negatively affecting patients’ survival. The latest group identified those SCLCs harbouring EGFR mutations (3.4%) deriving from NSCLC transformation. In this subgroup, an increased percentage of PI3KCA (5.6%) mutations were found; other mutational events were RBM10 loss of function and NFKBIA, NKX2-1, and CCNE1 gains of function or amplifications [19]. Recurrent rearrangements included RB1, NOTCH1, CREBBP, KMT2D and TP53 genes, thus assuming another inactivation event for these tumour suppressor genes. Although still very preliminary and requiring validation, these observations suggested that the evaluation of molecular profiling of SCLC could help clinicians to select the best therapeutic options for the patients.

Another contribution to the understanding of the biological background of SCLC has been recently provided by Sivapalan et al. [47], who collected tumour diagnostic biopsies and plasma samples from 33 SCLC patients before and during treatment course over a median follow-up of 11 months. Patients enrolled received chemotherapy or immunotherapy-containing regimes, both in first (n = 20) or further treatment lines (n = 13). Longitudinal ctDNA analyses of chromosomal (identified as plasma aneuploidy) and somatic sequence alterations were performed and then compared with tissue biopsies. Noteworthy, authors used white blood cell (WBCs) analyses in order to filter and remove germline and clonal haematopoiesis-related variants. A correspondence with a known SCLC molecular landscape was identified, with TP53 mutations as the most frequent molecular alteration along with other chromosomal rearrangements (i.e., across 1p or 5p arms). However, additional molecular abnormalities across different other genes, like PIK3CA, PALB2, EGFR, PTEN, BRAF, BRCA1-2 and KIT, were identified. A proof of concept analysis demonstrated a further correspondence between primary tumour features and ctDNA, thus confirming the possibility of defining a subclonal architecture by using ctDNA. Interestingly, sustained suppression of ctDNA levels correlated with both prolonged OS (HR = 0.09; 95% CI: 0.02–0.42, *p* = 0.002) and PFS (HR = 0.02, 95% CI: 0.00–0.16, *p* < 0.001), thus supporting the role of ctDNA as an early predictor of treatment efficacy and long-term clinical outcomes. [47].

## 3. Targeted Therapies in SCLC

SCLC has been considered “a graveyard for drug development” for a long time, with chemotherapy still representing the standard treatment across different lines of therapy. Differently from NSCLC, identifying targetable targets in SCLC has been challenging, also because most common molecular alterations regard either TP53 or RB1 genes that are currently considered pharmacologically untargetable. Several attempts have been made in the past with clinical trials investigating tailored inhibitors against different potential targets, such as mTOR, cKIT, MET, BCL-2, etc., overall failing to show any sign of activity in SCLC patients. Notwithstanding this, many target therapies are currently being investigated in this hard-to-treat and poor prognosis disease (Table 1).

### 3.1. CHK1 Inhibitors

SCLC cell lines harbour a higher level of both CHK1 gene and protein expression than NSCLC lines. Prexasertib, a CHK1 inhibitor, revealed strong anti-tumour activity in SCLC cell lines, SCLC syngeneic, genetically-engineered mouse (GEM) and chemo-resistant models [48]. The rational of targeting the CHK1/ATR axis in SCLC was confirmed with an independent preclinical study using ATR inhibitors demonstrating activity against SCLC in both in vitro and in vivo models. Promoting ATR through DNA damage leads to many downstream targets like CHK1, which stops cell cycle progression at the G2-M phase. A Phase II trial with Prexasertib in patients with an extended stage (ES)-SCLC was conducted to evaluate its efficacy. It was designed as a parallel-cohort phase II study of 105 mg/m^2^ prexasertib by IV administration. The drug was administered once every 14 days for patients who progressed after no more than two prior lines of therapy and had a platinum-sensitive (Cohort 1) or platinum-resistant/platinum-refractory (Cohort 2) disease. In Cohort 1 (n = 58), ORR was 5.2%; DCR, 31%; median PFS, 1.41 months (95% CI, 1.31–1.64), and median OS, 5.42 months (95% CI, 3.75–8.51). In Cohort 2 (n = 60), ORR was 0%; DCR, 20%; median PFS, 1.36 months (95% CI, 1.25–1.45), and median OS, 3.15 months (95% CI, 2.27–5.52). The most frequent all-grade, related, treatment-emergent adverse events were decreased neutrophil count (Cohort 1, 69.6%; Cohort 2, 73.3%), decreased platelet count (Cohort 1, 51.8%; Cohort 2, 50.0%), decreased white blood cell count (Cohort 1, 28.6%; Cohort 2, 40.0%), and anaemia (Cohort 1, 39.3%; Cohort 2, 28.3%). Eleven patients (19.6%) in Cohort 1 and one patient (1.7%) in Cohort 2 experienced grade ≥3 febrile neutropenia. Prexasertib did not demonstrate enough activity to be considered for future development as monotherapy in ED-SCLC [49].

### 3.2. PARPs (PARP Alone, PARPs plus CT, PARP plus ICIs, PARP plus anti DDR)

The anti-tumour activities of PARP inhibitors occur through different mechanisms, including trapping the enzyme to the single-strand DNA breaks (SSBs) by preventing the utilization of nicotinamide adenine dinucleotide (NAD), inhibiting poly ADP-ribosylation (PARylation), as well as binding of PARP to the DNA. Different studies tested PARPs inhibitors either as a single agent or in combination with other treatments. As their single-agent activity is limited, a series of clinical studies examined various combinations of PARP inhibitors with chemotherapy, radiation, and targeted therapies to increase their therapeutic benefit in this hard-to-treat disease. Owonikoko et al. tested the combination of veliparib with cisplatin (75 mg/m^2^) and etoposide (100 mg/m^2^ on days 1–3) in phase I/II randomized clinical trial (ECOG-ACRIN 2511), including patients with ES-SCLC. Patients with ES-SCLC, stratified by sex and serum lactate dehydrogenase levels, were randomly assigned to receive four three-week cycles of cisplatin-etoposide (CE) (75 mg/m^2^ intravenously on day 1 and 100 mg/m^2^ on days 1 through 3) along with veliparib (100 mg orally twice per day on days 1 through 7) or placebo (CE+P). The primary endpoint was PFS. The respective median PFS for the CE+V arm vs. the CE+P arm was 6.1 vs. 5.5 months (unstratified HR 0.75 [one-sided *p* = 0.06]; stratified HR, 0.63 [one-sided *p* = 0.01]), favouring CE+V. The mOS was 10.3 vs. 8.9 months (stratified HR, 0.83; 80% CI, 0.64 to 1.07; one-sided *p* = 0.17) for the CE+V and CE+P arms, respectively. The ORR was 71.9% vs. 65.6% (two-sided *p* = 0.57) for CE+V and CE+P, respectively. The following grade ≥ 3 haematology toxicities were more frequent in the CE+V arm than the CE+P arm: CD4 lymphopenia (8% vs. 0%; *p* = 0.06) and neutropenia (49% vs. 32%; *p* = 0.08), but treatment delivery was comparable. The addition of veliparib to frontline chemotherapy showed a signal of efficacy in patients with ES-SCLC, and the study met its prespecified end point [50].

Two phase II studies in relapsed SCLC patients evaluated the combination of temozolomide (TMZ) and PARP inhibition. Pietanza et al. performed a randomized, double-blind, placebo-controlled study of either veliparib (40 mg twice daily, days 1 to 7) or placebo and TMZ (150–200 mg/m^2^/day, days 1 to 5) on a 28-day cycle [51]. As a primary endpoint, the study had four-month PFS, with no significant differences observed between TMZ/veliparib (36%) and TMZ/placebo (27%, *p* = 0.19). Median PFS was 3.8 and 2.0 months (log-rank *p* = 0.39, HR 0.84; 95% CI: 0.56 to 1.25) for the TMZ/veliparib and TMZ/placebo arms, respectively. OS was also similar between the two arms. Instead, ORR has been shown to be higher for the combination of TMZ/veliparib (39%) vs. TMZ/placebo (14%) in both platinum-sensitive and platinum-refractory patients.

Using another PARP inhibitor, olaparib, in combination with TMZ, Farago et al. performed a phase I/II study in relapsed SCLC [52]. At the recommended phase II dose of olaparib (200 mg twice daily, day 1–7) and TMZ (75 mg/m^2^, day 1–7 of 21 days cycle), the ORR was 41%, with a median duration of response of 5.3 months. Across all dose levels, PFS was 4.2 months (95% CI, 2.8 to 5.7) with a median OS of 8.5 months (95% CI, 5.1 to 11.3). Another phase II study with continuous talazoparib associated with intermittent low-dose TMZ (NCT03672773) in relapsed/refractory SCLC is currently ongoing.

PARP inhibitors activity was also studied in combination with immunotherapy, based on a potential synergistic activity between such different approaches. A phase II trial in relapsed SCLC combining durvalumab 1500 mg every four weeks with olaparib 300 mg twice a day demonstrated an ORR of 10.5% (two patients out of nineteen) [53]. Similar results have been recently reported from the phase I/II multicentre open-label and single-arm basket MEDIOLA trial. From May 2016 to December 2016, 40 patients with limited or extended relapsing SCLC were enrolled. They received olaparib monotherapy (300 mg twice daily) for four weeks, followed by a combined treatment of olaparib (300 mg twice daily) and durvalumab 1500 mg iv administered every four weeks. ORR resulted in 10.5% (95% CI: 2.9–24.8). Meanwhile, mPFS was 2.4 months (95% CI: 0.9–3.0), and OS resulted in 7.6 months (95% CI: 5.6–8.8). Even if the study failed to reach the primary endpoint of disease control rate at 12 weeks (28.9%), one patient achieved a complete response and three a partial response. Grade 3 or higher adverse events were reported in 32 patients (80%), with anaemia (40%) and lymphopenia (12.5%) being the most frequent. These data suggested a limited activity of PARP inhibitors in SCLC patients, while additional studies are investigating the potential role of this therapeutic strategy in selected populations [54].

### 3.3. ATM/ATR Inhibitors

Another interesting approach might be the combination of ATM/ATR inhibitors together with the topoisomerase I inhibitor topotecan. A phase 2 trial was designed with a combination of Berzosertib (M6620), an ATP-competitive ATR inhibitor, and topotecan in SCLC patients who had relapsed after at least one prior chemotherapy. The primary endpoint was ORR. M6620 (210 mg/m^2^ intravenously on days 2 and 5) was administered concurrently with topotecan (1.25 mg/m^2^ intravenously on days 1 through 5) in 21-day cycles. A total of 26 patients were enrolled, and all of them had evidence of disease progression before study participation. Seven of 16 (43.8%) patients showed a partial response (PRs) in the first stage, permitting the continuation of enrolment in the second stage. In the overall study, 9 of 25 patients (36.0%, 95% CI: 18.0–57.5) obtained a confirmed partial response, reaching the primary endpoint for a response. Most patients (17/25 patients; 68.0%) obtained tumour regressions. After a median potential follow-up of 20.7 months, the median PFS was 4.8 months (95% CI: 2.8–7.4). The PFS at 4 and 6 months was 60.0% (38.4–76.1) and 36.0% (18.2–54.2), respectively. The median OS was 8.5 months (5.6–13.6), and OS at 6 and 12 months was 68.0% (46.1–82.5) and 32.0% (15.2–50.2), respectively. Responses were achieved in patients with both platinum-sensitive and platinum-resistant disease. These trial results provide evidence to support the strategy of a mixed ATR and TOP1 inhibition in order to empower the topotecan efficacy in SCLC patients [55].

### 3.4. AURKA/B Inhibitors

Inhibition of Aurora kinase A or B arrests the proliferation and growth of both in vitro and in vivo SCLC models [56]. In a recently reported clinical trial, an aurora kinase A inhibitor, alisertib, combined with paclitaxel, had significantly improved PFS compared to paclitaxel alone in patients with cMYC-positive SCLC. The efficacy of targeting AURKA was studied in a randomized phase II study of paclitaxel plus alisertib vs. paclitaxel plus placebo as second-line therapy. In this double-blind study, patients affected by relapsed or refractory SCLC were stratified considering the relapse pattern (sensitive vs. resistant or refractory) as well as the presence of brain metastases and randomized 1:1 to alisertib/paclitaxel or placebo plus paclitaxel. A total of 178 patients were enrolled (89 in each arm). The median PFS was 3.32 months with alisertib/paclitaxel vs. 2.17 months with placebo/paclitaxel (HR = 0.77), thus confirming a promising activity of alisertib/paclitaxel in relapsed or refractory SCLC [57].

### 3.5. DLL3 Inhibitors

In SCLC, there are common inactivating mutations in the primary Notch family members and overexpression of a key negative regulator of Notch signalling known as delta-like protein 3 (DLL3) was found in the majority of SCLC tumours [58]. Initial clinical evaluation of an anti-DLL3 antibody-drug conjugate rovalpituzumab tesirine (Rova-T) had promising activity, although this agent was comprised of several toxicities [59]. Rovalpituzumab tesirine (Rova-T) has the structure of an antibody-drug conjugate containing a DLL3-targeting antibody joined to a cytotoxic agent, pyrrolobenzodiazepine. The efficacy and safety of Rova-T compared with topotecan as second-line therapy were evaluated in patients with SCLC expressing high levels of DLL3 (DLL3-high). The TAHOE study was an open-label, two-to-one randomized, phase 3 study comparing Rova-T with topotecan. The setting of this study was the second-line therapy in DLL3-high advanced or metastatic SCLC. Rova-T (0.3 mg/kg) was given intravenously on day 1 of a 42-day cycle for two cycles, with two additional cycles available for specific patients. Topotecan (1.5 mg/m^2^) was administered intravenously on days 1 to 5 of a 21-day cycle. Patients randomized to Rova-T (n = 296) and topotecan (n = 148) were included in the efficacy analyses. The median OS was 6.3 months (95% CI: 5.6–7.3) in the Rova-T arm and 8.6 months (95% CI: 7.7–10.1) in the topotecan arm (HR, 1.46 [95% CI: 1.17–1.82]). An independent data monitoring committee stated that enrolment had to be discontinued because of the shorter OS observed with Rova-T compared with topotecan. Safety profiles for both drugs were not different from previous reports. Compared with topotecan, the current standard of care for second-line chemotherapy, Rova-T demonstrated an inferior OS and higher rates of side effects. These effects were represented by serous effusions, photosensitivity reactions, and peripheral oedema. Despite this failure, other trials are currently evaluating anti-DLL3 efficacy in SCLC.

Tarlatamab, a bispecific T-cell engager molecule (BiTE), in patients with relapsed/refractory SCLC, was evaluated in a phase 1 study. The primary end point was safety. Secondary end points included antitumor activity by modified RECIST 1.1, overall survival, and pharmacokinetics. By 19 July 2022, 107 patients received tarlatamab within both dose exploration (0.003 to 100 mg; n = 73) and expansion (100 mg; n = 34) cohorts. Median prior lines of anti-cancer therapy achieved by patients were 2 (range, 1–6); 49.5% received anti-programmed death-1/programmed death ligand-1 therapy. Any-grade treatment-related adverse events occurred in 97 patients (90.7%) and grade ≥ 3 in 33 patients (30.8%). One patient (1%) experienced grade 5 pneumonitis. Cytokine release syndrome was the most common treatment-related adverse event, occurring in 56 patients (52%), including grade 3 in one patient (1%). The maximum tolerated dose was not reached. The objective response rate was 23.4% (95% CI, 15.7 to 32.5), including two complete and 23 partial responses. The median duration of response was 12.3 months (95% CI, 6.6 to 14.9). The disease control rate was 51.4% (95% CI, 41.5 to 61.2). The median PFS and OS were 3.7 months (95% CI, 2.1 to 5.4) and 13.2 months (95% CI, 10.5 to not reached), respectively. Exploratory analysis suggests that selecting for increased DLL3 expression can result in increased clinical benefit [60]. In patients with heavily pre-treated SCLC, tarlatamab showed manageable safety with promising response durability. Further evaluation of this promising molecule is ongoing in the context of prospective randomized clinical studies.

### 3.6. RNA Polymerase II Inhibitors

Lurbinectedin, a DNA binding agent that seems to work as a selective inhibitor of RNA polymerase II transcription, demonstrated substantial activity against SCLC [61]. This drug induces selective degradation of RNA pol. II leading to apoptosis in tumour cells. The evidence of lurbinectedin activity in SCLC derives from a cohort of a single-arm, open-label, phase II basket trial conducted by Trigo et al. [62]. The authors enrolled 105 patients with advanced SCLC pre-treated with only one previous line of treatment (IO was allowed alone or in combination with CHT) and Eastern Cooperative Oncology Group (ECOG) performance status of two or lower. According to the investigator’s assessment, after a median follow-up of 17.1 months, the study reached its primary endpoint with a RR of 35.2% (95% CI: 26.2–45.2) in the entire cohort. In a pre-planned conducted analysis, the overall responses were higher in patients with sensitive disease compared with resistant disease. Of note, 60.9% and 27.1% of patients were still alive after one and two years, respectively. When considered together, these data are very important in terms of response and survival, if compared with historical controls, in both groups of patients with resistant and sensitive diseases. Furthermore, lurbinectedin had a good safety profile with manageable toxicity. After the positive results of this phase II study, on June 2020, lurbinectedin received approval from the FDA for patients with SCLC in progression on or after platinum-based CT and has recently been granted orphan drug status by the European Medicine Agency (EMA). Lurbinectidin was later evaluated in a phase III trial [63]. Atlantis study is an open-label, randomized, multicenter phase III trial testing the second-line efficacy of the combination of lurbinectedin and doxorubicin compared to the investigator’s choice of CT with CAV (cyclophosphamide/doxorubicin/vincristine) or topotecan. In this study were enrolled pre-treated patients with histologically confirmed diagnoses of limited or ED SCLC whose disease progressed after one prior platinum-containing line. Its first endpoint, the OS, was not reached.

In conclusion, lurbinectedin has demonstrated good activity as a single agent in second-line therapy of SCLC, to a large extent in platinum-sensitive patients, but failed to exhibit an improvement in OS when combined with doxorubicin compared with CAV or topotecan. Although the primary endpoint of OS in the phase III study was not reached, lurbinectedin plus doxorubicin showed a good safety profile. Lurbinectedin is a treatment option for patients progressing on or after first-line platinum-based ChT [13,64].

### 3.7. VEGF Inhibitors

Several randomized trials tested the anti-VEGF monoclonal antibody, bevacizumab, in combination with standard chemotherapy in SCLC patients, showing poor results and no clear survival benefits [65,66]. A randomized phase III, open-label, multicentre clinical trial enrolled 205 patients with ED-SCLC, investigating bevacizumab in combination with etoposide and cisplatin in the first line. At a median follow-up of 34.9 months, an improvement of median OS (9.8 vs. 8.9 months; HR = 0.78), 1-year survival rates (37% vs. 25%) and objective response (58.4% vs. 55.3%) has been observed in favour of bevacizumab-treated patients.

Sorafenib combined with chemotherapy was reported to have significant toxicity and low efficacy in a phase 2 trial [67]. A total of 18 patients were enrolled, with 17 evaluable patients. One patient had a complete response, seven patients had a partial response (overall response rate of 47%), and one patient had stable disease. Median OS was 7.4 months, and one-year survival was 25%. The most common treatment-related adverse events included fatigue, anorexia, rash, diarrhoea, neutropenia and weight loss. Grade 5 gastrointestinal bleeding, pulmonary haemorrhage and neutropenia occurred in one patient (6%) each. Accrual was halted on the basis of the safety profile as well as preliminary efficacy data. The combination of platinum-based chemotherapy and sorafenib has significant toxicity at current dose levels and is associated with disappointing efficacy data.

Thalidomide is another anti-angiogenic drug that was evaluated in SCLC [68]. In a phase 3 trial, thalidomide combined with chemotherapy did not improve survival in SCLC patients with limited disease or extensive disease [69]. Thalidomide was also investigated both in combination with carboplatin-etoposide and as maintenance therapy in patients with untreated SCLC. Median progression-free and overall survival were 8.3 months and 10.1 months, respectively. One-year survival was 40%, and the one-year progression-free survival was 36%. The ORR was 68% (95% CI 46–85%), with four complete remissions (20%) and 13 partial remissions (48%). No increase in chemotherapy-related toxicity was observed. Thalidomide was well-tolerated, and the median time on thalidomide treatment was 7.6 months.

Differently from other VEGF inhibitors, apatinib, a selective target of VEGFR2, demonstrated good results in previous studies and also in SCLC settings. A phase II trial showed acceptable toxicity in pre-treated patients receiving apatinib. Forty patients were enrolled. At the data cut-off time (15 November 2018), the median follow-up was 7.4 months; no patients remained on treatment, and five were still in follow-up. An objective response was achieved in 7 of 40 patients (17.5%) in the intention-to-treat population and 7 of 38 patients (18.4%) in the per-protocol population. The median PFS and OS were 3.0 months and 5·8 months, respectively. The most commonly observed grade 3 or greater treatment-related adverse events were hypertension, hand-foot syndrome, and increased L-gamma-glutamyltransferase [70]. Apatinib exhibited efficacy and an acceptable safety profile in previously heavily-treated ES-SCLC patients. Further exploration of apatinib in phase III trials is warranted.

Similarly to apatinib, another angiogenic multikinase inhibitor, anlotinib, exhibiting activity against VEGFR 1-2-3, FRGR 1-4, PDGFR a/b and c-Kit, has shown encouraging results. From 2017 to 2018, a prospective randomised, double-blind trial was conducted to evaluate its efficacy (versus placebo) in patients affected by SCLC failing at least two prior lines of treatment. The study demonstrated a PFS advantage over placebo: 4.1 months (95% CI: 2.8–4.2) vs. 0.7 months (95% CI: 0.7–0.8), with an acceptable toxicity profile. To date, several clinical trials are currently ongoing to evaluate its efficacy and safety in different settings [71].

### 3.8. EZH2 and LSD1 (Epigenetic) Inhibitors

Since the human epigenome could be visualized using next-generation sequencing, the role of epigenetic processes in SCLC could be understood [63]. The most promising epigenetic regulatory proteins are enhancers of zeste homolog 2 (EZH2) and lysine-specific demethylase 1A (LSD1). Both of them are now being tested in SCLC clinical trials. Respectively two promising drugs against EZH2 and LSD1 tested in SCLC are Tazemetostat and GSK2879552. EZH2 itself is a common target of deregulated expression in cancers. Aberrant EZH2 expression in cancers is due to genetic, transcriptional, post-transcriptional, and post-translational modifications [72]. EZH2 inhibitors are mostly tested with platinum-based compounds but are also being explored in the context of combination regimens, including docetaxel, etoposide, temozolomide (chemotherapy), PD-L1 and PD-1 inhibitors, antiandrogens, PARP and HDAC inhibitors. These are expected to increase the effects of EZH2-targeted therapy. Gardner et al. showed that chemoresistance to cisplatin and etoposide in SCLC is partially due to the suppression of SLFN11, a protein with the role of inhibiting DNA replication and promoting cell death after DNA damage. The authors showed that EZH2 interacting with SLFN11 promotes chemoresistance [73]. Elements that definitely demonstrate the concrete activity of anti-EZH2 in SCLC are still immature and under investigation.

**Table 1:** Ongoing clinical trials of targeted therapies in SCLC (from clinicaltrials.gov, latest access on 30 March 2023). NA: not applicable.

Molecular Target	Trial Identifier	Status	Phase	Drugs Tested	Main Setting
**PARPs**	NCT04826341	Recruiting	I/II	Sacituzumab-Govitecan plus Berzosertib	Recurrent histologically or cytologically confirmed SCLC after at least one prior platinum-based therapy.
	NCT04728230	Recruiting	I/II	Carboplatin plus etoposide plus durvalumab plus olaparib and/or radiation therapy	No prior systemic therapy for ES-SCLC, including, but not limited to, chemotherapy, PARP inhibitor, and PD-1/PD-L1 checkpoint inhibitors.
	NCT04701307	Active, not recruiting	II	Niraparib and dostarlimab	Second-line therapy.
	NCT03532880	Active, not recruiting	I	Olaparib and low-dose radiotherapy	Completion of induction chemotherapy for ES-SCLC with a minimum of 4 and no more than 6 cycles of a platinum agent and etoposide within 8 weeks of trial initiation and no progression of the disease.
	NCT03672773	Active, not recruiting	II	Talazoparib and low-dose temozolomide	Relapsed or refractory ES-SCLC.
	NCT05411679	Not yet recruiting	II	EP0057, in combination with olaparib	Two prior lines of systemic therapy for ES-SCLC, providing patients have not received irinotecan in the second-line setting or one prior line of therapy if considered to be unwilling or unsuitable for the current standard of care treatment options.
	NCT03923270	Active, not recruiting	I	Radiotherapy plus durvalumab alone vs. durvalumab combinations (tremelimumab or olaparib)	Maintenance after platinum-based first-line chemotherapy for ES-SCLC.
	NCT04209595	Active, not recruiting	I/II	PLX038 (PEGylated SN38) and rucaparib	Progressed on or after standard first-line systemic chemotherapy for SCLC.
	NCT03227016	Unknown	I	Veliparib in combination with topotecan	Refractory to prior chemotherapy ES-SCLC.
	NCT03830918	Recruiting	II	Niraparib, temozolomide and atezolizumab	A complete or partial response to platinum-based first-line chemotherapy in ES-SCLC.
	NCT04334941	Active, not recruiting	II	Atezolizumab and talazoparib	ES-SCLC patients with SLFN11-positive biomarkers randomised to atezolizumab or atezolizumab plus talazoparib as maintenance therapy.
	NCT04434482	Recruiting	I	IMP4297, in combination with temozolomide	Second-line therapy for ES-SCLC.
	NCT05002868	Recruiting	I	RP12146	Second-line therapy for ES-SCLC in case of documented deleterious mutations of specified HRR genes.
	NCT02769962	Recruiting	I/II	EP0057 and olaparib	Second-line therapy for ES-SCLC.
	NCT04644068	Recruiting	I/II	AZD5305 as monotherapy and in combination with anti-cancer agents	Patients with progressive cancer (i.e., ES-SCLC) must not have received prior therapy with a PARPi-based regimen.
	NCT03958045	Active, not recruiting	II	Rucaparib and nivolumab	Platinum-Sensitive ES-SCLC patients as maintenance after induction therapy with the platinum doublet.
	NCT02734004	Active, not recruiting	I/II	MEDI4736 in combination with olaparib	Confirmed progressive ES-SCLC.
	NCT02498613	Active, not recruiting	II	Cediranib in combination with olaparib	Second-line therapy for ES-SCLC.
	NCT04400188	Active, not recruiting	I/II	Fluzoparib (SHR-3162) and temozolomide with or without SHR-1316	Second-line therapy for ES-SCLC.
	NCT04659785	Unknown	I/II	Fluzoparib combined with apatinib	Second-line therapy for ES-SCLC.
**ATM**	NCT04939662	Recruiting	II	Olaparib and bevacizumab	Relapsed ES-SCLC with ATM deficiency, SLFN11 positive or POU2F3 positive or HR gene mutation.
	NCT04514497	Recruiting	I	Addition of BAY 1895344 to the usual chemotherapy	Second-line therapy for ES-SCLC.
	NCT04768296	Active, not recruiting		Berzosertib plus topotecan	Relapsed platinum-resistant ES-SCLC.
	NCT02487095	Active, not recruiting	I/II	Topotecan with berzosertib	Second-line therapy for ES-SCLC.
**ATR**	NCT04802174	Recruiting	I/II	Lurbinectedin with berzosertib	Second-line therapy for ES-SCLC.
	NCT03896503	Active, not recruiting	II	Topotecan with berzosertib	Relapsed ES-SCLC.
	NCT02595931	Active, not recruiting	I	Berzosertib and irinotecan hydrochloride	ES-SCLC refractory to standard therapy.
**AURKB**	NCT03216343	Recruiting	I	Chiauranib	At least 2 different systemic chemotherapy regimens (contained platinum-based regimen) and progressed or relapsed ES-SCLC.
	NCT04830813	Recruiting	III	Chiauranib capsule	At least 2 different systemic chemotherapy regimens (contained platinum-based regimen) and progressed or relapsed ES-SCLC.
**DDL3**	NCT05507593	Recruiting	I	DLL3-CAR-NK cells	Relapsed and refractory ES-SCLC and disease progression within 6 months after the last-line treatment.
	NCT05680922	Recruiting	I	DLL3-Directed chimeric antigen receptor T-cells	ES-SCLC after progression to at least one prior line of standard treatment or in case of insufficient response, and for those for whom standard treatment is intolerable or unlikely to confer significant clinical benefit.
	NCT04429087	Recruiting	I	BI 764532	ES-SCLC DDL3+ after at least one line of chemotherapy that should include platinum.
	NCT04471727	Recruiting	I/II	HPN328 monotherapy or with atezolizumab	Relapsed/refractory following at least 1 prior line of systemic therapy that included platinum-based chemotherapy. Expression of DLL3 required.
	NCT03319940	Recruiting	I	AMG 757 monotherapy, in combination with anti-PD1 therapy and with additional cytokine release syndrome (CRS) mitigation strategies	Progressed or recurred following platinum-based regimen ES-SCLC.
	NCT05652686	Recruiting	I	PT217	At least one line of platinum-based chemotherapy with or without ICIs for ES-SCLC patients.
	NCT04885998	Active, not recruiting	I	AMG 757 and AMG 404	Second-line therapy for ES-SCLC.
	NCT05060016	Recruiting	II	AMG 757	ES-SCLC patients who progressed or recurred following one platinum-based regimen and at least one other prior line of therapy.
	NCT05361395	Recruiting	I	AMG 757 in combination with carboplatin, etoposide and PD-L1 inhibitor	ES-SCLC and no prior systemic treatments for the extended stage.
**RNA pol. II**	NCT05091567	Recruiting	III	Maintenance lurbinectedin in combination with atezolizumab	Ongoing response or stable disease per RECIST 1.1 after 4 cycles of induction therapy for ES-SCLC.
	NCT04358237	Active, not recruiting	I/II	Lurbinectedin combined with pembrolizumab	Second-line therapy for ES-SCLC.
	NCT04253145	Unknown	I	Lurbinectedin and atezolizumab	Progression to first-line platinum-based chemotherapy for ES-SCLC.
**EZH2**	NCT03879798	Active, not recruiting	I/II	DS-3201b plus irinotecan	Second-line therapy for ES-SCLC.
	NCT03460977	Closed to enrolment for SCLC	I	PF-06821497	Relapsed or refractory ES-SCLC.
**LSD1**	NCT05191797	Recruiting	I/II	Bomedemstat and maintenance immunotherapy	Maintenance immunotherapy for patients with newly diagnosed ES-SCLC.
	NCT05268666	Recruiting	I/II	JBI-802	ES-SCLC have received ≤2 prior regimens, which must have included ICIs and platinum-based chemotherapy.
	NCT04350463	Active, not recruiting	II	CC-90011, in combination with nivolumab	ES-SCLC progressing after 1 or 2 lines of therapies.
**CDK7**	NCT04247126	Active, not recruiting	I	SY-5609 plus gemcitabine	Advanced solid tumours for which standard curative or palliative measures do not exist or are no longer effective.
**VEGFR2**	NCT04683198	Not yet recruiting	II	Camrelizumab combined with apatinib, carboplatin and etoposide	First-line ES-SCLC.
	NCT04490421	Unknown	III	Camrelizumab combined with apatinib, etoposide and cisplatin	First-line Treatment SCLC.
	NCT05001412	Recruiting	I	Chemotherapy combined With camrelizumab and apatinib	First-line treatment of ES-SCLC; limited SCLC patients have received radiotherapy and chemotherapy for more than 6 months.
	NCT04453930	Recruiting	II	Camrelizumab chemotherapy (irinotecan plus platinum) and with apatinib	Untreated ES-SCLC.
	NCT02875457	Not yet recruiting	III	Maintenance with apatinib	ES-SCLC after being combined with etoposide/cisplatin.
	NCT04901754	Unknown	II	Camrelizumab plus apatinib as Maintenance	ES-SCLC after first-line standard chemotherapy.
	NCT03389087	Unknown	II	Apatinib and oral etoposide	Third-line therapy for ES-SCLC.
**VEGF** **FGFR** **c-kit**	NCT04684017	Unknown	II	Anlotinib Plus etoposide and carboplatin	First-line therapy for ES-SCLC.
	NCT05027100	Recruiting	NA	Tislelizumab combined with anlotinib and 2-cycles of irinotecan	Second-line therapy for ES-SCLC.
	NCT04620837	Recruiting		Tislelizumab in combination with anlotinib	Maintenance after first-line chemotherapy for ES-SCLC.
	NCT03780283	Unknown	II	Anlotinib	Maintenance after first-line chemotherapy for ES-SCLC.
	NCT03700359	Unknown	II	Lobaplatin/etoposide with or without anlotinib	First-line therapy for ES-SCLC.
	NCT04055792	Unknown	II	Anlotinib combined with sintilimab vs. anlotinib alone	Third-line or beyond chemotherapy for ES-SCLC.
	NCT04660097	Recruiting	II	Alotinib plus durvalumab-platinum-etoposide	First-line therapy for ES-SCLC.
	NCT03890055	Unknown	IV	Platinum/etoposide and anlotinib	First-line therapy for ES-SCLC.
	NCT04967625	Not yet recruiting	II	Sintilimab combined with anlotinib	Second-line therapy for ES-SCLC.
	NCT05001971	Recruiting	II	Anlotinib plus penpulimab	Second-line therapy for ES-SCLC.
	NCT03781869	Unknown	II	Anlotinib	Maintenance after first-line therapy for ES-SCLC.
	NCT04363255	Not yet recruiting	II	Platinum/etoposide and toripalimab combined with anlotinib	First-line therapy for ES-SCLC.
	NCT04192682	Unknown	II/III	Anlotinib combined with sintilimab	Second-line therapy for ES-SCLC.
	NCT04731909	Recruiting	NA	Platinum/etoposide and toripalimab combined With anlotinib	First-line therapy for ES-SCLC.
	NCT04882033	Recruiting	I	Platinum/etoposide/radiotherapy and anlotinib	First-line therapy for LS-SCLC.
	NCT03732846	Unknown	II	Anlotinib	Previous two or more lines or therapy for ES-SCLC.
	NCT04675697	Unknown	II	Platiinum/etoposide and anlotinib	First-line therapy for ES-SLCL.
	NCT04985851	Recruiting	NA	Durvalumab plus anlotinib	Maintenance after first-line chemoimmunotherapy for ES-SCLC.
	NCT04073550	Unknown	III	Anlotinib/placebo and topotecan	Progression disease in ES-SCLC.
	NCT04933175	Not yet recruiting	II	Fluzopalil in combination with anlotinib	Second-line therapy for ES-SCLC.
	NCT04757779	Recruiting	II	Anlotinib with irinotecan or docetaxel	Second-line therapy for ES-SCLC.
	NCT04234607	Unknown	III	TQB2450 or placebo combined with anlotinib, etoposide and carboplatin	First-line therapy for ES-SLCL.
	NCT03841136	Unknown	II	Anlotinib combined with etoposide and platinum	First-line therapy for ES-SLCL.
	NCT04165330	Recruiting	I/II	Anlotinib in combination with nivolumab	Second-line therapy for ES-SCLC.

## 4. Discussion

The clinical management of small cell lung cancer still represents a major challenge for thoracic oncologists. Over the last few decades, we have witnessed several steps forward in the clinical management of NSCLC in terms of both early diagnoses and therapeutic improvements as the introduction of targeted therapies and immunotherapy. Unlikely, in SCLC, there have been very few therapeutic advances impacting patients’ survival. Since the recent introduction of ICIs in combination with platinum-chemo as a new first-line standard treatment [10,11], no other drugs represented a major breakthrough in this disease. Despite several new promising therapies currently under investigation, the therapeutic scenario for relapsing ED-SCLCs remains almost deserted.

Differently from NSCLC, there was historically limited knowledge about the molecular background of SCLC, but recent efforts have been made to clarify the molecular features of this disease, leading to the identification of key signalling pathways involved in tumourigenesis processes, which may serve as a potential target for clinical use [43]. Despite the large number of molecules tested and the numerous therapeutic failures, some targeted therapies have recently shown interesting preliminary results. Among the most attractive drugs currently under clinical investigation, there are some tyrosine kinase inhibitors, multiple receptor inhibitors, and antibodies drug-conjugates (ADC) or bispecific T-cell engager (BiTEs).

The history of DDL3 therapeutic targeting represents a paradigm of drug development evolution in this hard-to-treat disease, moving from the clinical failures of Rovalpituzumab tesirine, as emerged by the TRINITY and TAHOE randomized trials [59,74] to the recent advent of tarlatamab (AMG757), a BiTE molecule simultaneously targeting both CD3 and DDL3. Data from the phase 1 trial showed encouraging preliminary results in terms of ORR (23.4%) and median duration of response (12.3 months) with a tolerable safety profile in heavily pre-treated SCLC patients, with an exploratory analysis suggesting increased activity in those patients harbouring higher DDL3 expression. In the same setting, other DDL3 inhibitors have been investigated, including AMG 119, a DDL3-directed CART cell therapy, which has shown encouraging results in preclinical models and is currently being tested in phase I clinical trial (NCT03392064) [75].

Although SCLC is still considered a unique disease, recent evidence revealed that it is characterised by a high level of biological heterogeneity as well as different molecular subtypes. A large real-world clinical study led to the identification of new molecular patterns and recurrent mutational features. Particularly interesting in this regard is the hypothesis of an HPV-related SCLC origin in the case of TP53 and/or RB1 WT tumours; these have been intriguingly identified more frequently among younger or African ancestry patients. The same analysis identified recurrent alterations related to specific metastatic sites: i.e., PTEN mutations have been correlated to brain metastases (19.8% vs. 9.7%, *p* = 0.012). Meanwhile, chromosomal arm-level increases have been related to both brain and liver metastases. Further evidence with similar large cohort studies is thus needed to confirm these hypotheses in order to find stronger correlations between molecular landscape and clinical features and survival outcomes [19]. Recent data also revealed a high concordance rate between ctDNA and molecular tissue analysis in extensive-stage SCLC patients as well as a strict correlation between ctDNA level dynamic variation and therapeutic response to systemic antitumor treatments, suggesting the potential role of ctDNA as an early indicator of treatment efficacy for the clinical setting. However, how this can actually impact patients’ clinical management and outcomes is not already clear and further validations are needed in this setting.

In conclusion, recent efforts have offered new possibilities for implementing SCLC patients’ prognoses. A deeper understanding of the molecular background, along with the development of innovative targeted approaches, is driving a significant step forward in the clinical management of this disease, elucidating the therapeutic vulnerabilities of this disease and finally supporting the development of personalised strategies to be offered to our patients.

## Data Availability

Not applicable.
